# High variability in bodyweight is associated with an increased risk of atrial fibrillation in patients with type 2 diabetes mellitus: a nationwide cohort study

**DOI:** 10.1186/s12933-020-01059-8

**Published:** 2020-06-13

**Authors:** Hyun-Jung Lee, Eue-Keun Choi, Kyung-Do Han, Da Hye Kim, Euijae Lee, So-Ryoung Lee, Seil Oh, Gregory Y. H. Lip

**Affiliations:** 1 Department of Internal Medicine, Seoul National University Hospital, 101 Daehak-ro, Jongno-gu, Seoul, 03080 Republic of Korea; 2 Department of Statistics and Actuarial Science, Soongsil University, Seoul, Republic of Korea; 3 Liverpool Centre for Cardiovascular Science, University of Liverpool and Liverpool Heart and Chest Hospital, Liverpool, UK; 4 Aalborg Thrombosis Research Unit, Department of Clinical Medicine, Aalborg University, Aalborg, Denmark

**Keywords:** Diabetes mellitus, Bodyweight, Variability, Atrial fibrillation, Obesity

## Abstract

**Background:**

Bodyweight variability is a risk factor for atrial fibrillation (AF). We aimed to examine the relationship between bodyweight variability and the risk of AF in patients with type 2 diabetes mellitus (DM), and whether this relationship was affected by baseline body mass index (BMI), weight change, or advanced diabetic stage.

**Methods:**

A nationwide population-based cohort of 670,797 patients with type 2 DM from the Korean National Health Insurance Service database without a history of AF and with ≥ 3 measurements of bodyweight over a 5-year period were followed up for AF development. Intra-individual bodyweight variability was calculated using variability independent of mean, and high bodyweight variability was defined as the quintile with the highest variability with the lower four quintiles as reference.

**Results:**

During a median of 7.0 years of follow-up, 22,019 patients (3.3%) newly developed AF. After multivariate adjustment, those in the highest quintile of bodyweight variability showed a higher risk of incident AF (HR 1.16, 95% CI 1.12–1.20) compared to those in the lower 4 quintiles with reference bodyweight variability, irrespective of baseline BMI group and direction of overall weight change. This association was greater in magnitude in subjects with lower BMI, those on insulin, and those with a DM duration of greater than 5 years. In sensitivity analyses, high bodyweight variability was consistently associated with AF development using other indices of variability and adjusting for glycemic variability.

**Conclusions:**

High variability in bodyweight was associated with AF development, independently of traditional cardiovascular risk factors and baseline BMI. This association was stronger in underweight patients and with advanced diabetic stage. Weight fluctuation may interfere with the beneficial effects of weight loss and should be avoided when possible in weight control regimens for DM patients.

## Background

Atrial fibrillation (AF) is the most common sustained arrhythmia in the general population, and its prevalence is progressively increasing [1]. Diabetes mellitus (DM) and obesity are both established risk factors for AF [2, 3]. DM and obesity often coexist, and have synergistic effects on the development of AF [4, 5]. Interventions for weight loss lower the risk of AF development or AF burden [6–8], and weight loss is recommended in overweight and obese DM patients [9]. However, attempts at weight loss are often accompanied by fluctuations in weight, with 80% of individuals who intentionally lose > 10% of bodyweight often regaining that initial weight loss within 1 year [10].

Bodyweight fluctuation has been associated with increased cardiovascular events and mortality in the general population and patients with coronary artery disease [11, 12] as well as in DM patients [13, 14]. Bodyweight variability has also been associated with an increased risk of AF in the general population [15], but this has not been studied with a specific focus on the diabetic population.

The purpose of this study was to examine the relationship between bodyweight variability and the risk of AF in patients with type 2 DM, and whether this relationship was affected by baseline body mass index, weight change, or advanced diabetic stage.

## Methods

### Study population

This study utilized a nationwide population-based claims database from the National Health Insurance Service, which provides health care insurance coverage and regular health examinations for the entire Korean population, and the details of this database have been described previously [15, 16]. Data is available to researchers approved by their institutional review boards on request at the National Health Insurance Sharing Service hompage (nhiss.nhis.or.kr). The general population is required to undergo government-covered health examinations annually or biennially depending on the occupation, and the inspection rate is around three-fourths (76.1% from a total 17,633,406 subjects scheduled to take the 2015 health examinations). Patient demographics, all data on healthcare usage and prescriptions, health examination results, and mortality information are included in the database.

**Fig. 1:**
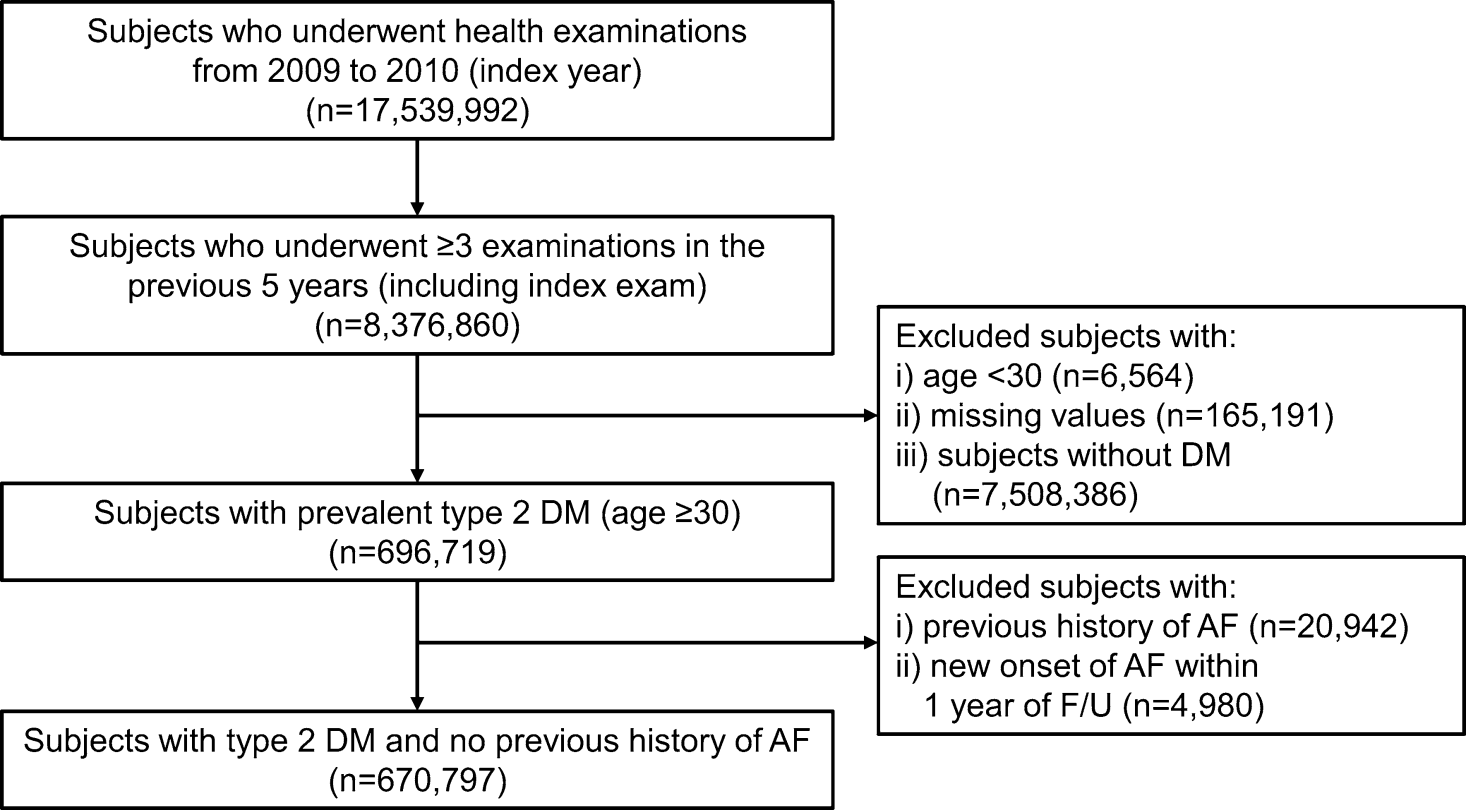
Study inclusion flow. DM, diabetes mellitus; AF, atrial fibrillation; Q, quintile

The study inclusion flow is shown in Fig. 1. From the database, 17,539,992 subjects who underwent health examinations in 2009 and 2010 were identified. Subjects who underwent ≥ 3 examinations in the previous 5 years (from 2004–2005), including the index exam, were included (n = 8,376,860). We included adult patients with prevalent type 2 DM (age ≥ 30) regardless of the onset, and excluded subjects with missing parameters in the health examination or with a previous history of AF. Subjects under the age of 30 were not included as weight gain is part of the natural process of growth in children. We ascertained outcome events after a lag of 1 year, and those with outcome events within 1 year were excluded (n = 4980). Finally, a total of 670,797 subjects with type 2 DM, and no previous history of AF were included in the study.

The index date was the date of the last health examination, and follow-up was until AF was newly diagnosed, or censoring by death, loss of health insurance qualification, or end of study (December 31, 2017). Baseline characteristics and health examination results were those of the index year. This study was approved by the institutional review board of Seoul National University Hospital (E-1811-130-987), and informed consent was waived.

### Definitions of diseases and outcome

Patients with DM were defined as follows: (i) having at least one claim per year for a prescription of anti-diabetic medication under *International Statistical Classification of Diseases, Tenth Revision* (ICD-10) codes E11–14 from the insurance claims data, or (ii) having a fasting plasma glucose ≥ 126 mg/dL in the health examination without a prescription of anti-diabetic medication [4, 17, 18]. Anti-diabetic medications included sulfonylureas, metformin, DPP4 inhibitors, thiazolidinediones, alpha-glucosidase inhibitors, meglitinides, and insulins. Patients with type 1 DM (ICD-10 code E10) were excluded from this study. Comorbidities were defined using ICD-10 diagnosis codes with health care usage and medication, or health examination results, as in the previous studies [15, 19, 20], and are described in Additional file 1: Table S1. Definitions of lifestyle behaviors are described in Additional file 1: Table S2. Medications were assessed at the index year, and duration of DM was measured from the first diagnosis of DM up to the index date.

The primary endpoint was newly-diagnosed nonvalvular AF. This was defined as the first diagnosis of AF during admission or at the outpatient clinic (ICD-10 code I48) in the claims database, with the exclusion of rheumatic mitral stenosis or prosthetic heart valves [15, 21].

### Definition of bodyweight variability

Intra-individual bodyweight variability between visits was calculated using variability independent of the mean (VIM). VIM is a measure of variability designed not to correlate with mean levels and is calculated as SD/mean^χ^, where χ is derived from fitting curves by nonlinear regression analysis to SD = constant*mean^χ^ so that there is no correlation with the mean [11]. Other measures of variability including standard deviation (SD), coefficient of variation (CV), average successive variability (ASV) [15, 22] were used for sensitivity analyses.

Body mass index (BMI) was calculated as the weight (in kilograms) divided by the height (in meters squared). Subjects were categorized into 5 groups according to BMI: underweight (< 18.5 kg/m^2^), normal weight (18.5–22.9 kg/m^2^), overweight (23–24.9 kg/m^2^), obese stage I (25–29.9 kg/m^2^), and obese stage II (≥ 30 kg/m^2^), according to World Health Organization recommendations for Asians [23]. Overall bodyweight change was the change between the first and last health examinations. Subjects were categorized according to total bodyweight change into 3 groups: weight loss (weight decrease ≥ − 5%), stable weight (weight change within 5%), and weight gain (weight increase ≥ 5%).

### Statistical analysis

Continuous variables are presented as mean ± SD, and categorical variables as n (%). The incidence rates of AF are presented per 1000 person-years. Multivariate Cox regression analysis was used to assess the risk of developing AF associated with bodyweight variability, with adjustment for baseline BMI, age, sex, smoking, drinking, exercise, low income, hypertension, dyslipidemia, number of oral anti-diabetic medication, insulin use, duration of DM, and fasting glucose. The proportional hazards assumption was valid for all predictors in graphic evaluation of log–log plots. High bodyweight variability was defined from the cut-off that AF risk started to increase, after evaluation of the relationship between bodyweight variability and risk of AF development. Analyses were performed to assess the relationship between high bodyweight variability and AF risk based on baseline BMI categories and overall bodyweight change, using Cox regression models with interaction terms. Subgroup analyses were conducted for age strata, sex, presence of comorbidities, and measures of DM severity. Sensitivity analyses were performed using other indices of variability (SD, CV, ASV), and with adjustment for glycemic variability. Statistical analyses were performed using SAS version 9.4 (SAS Institute Inc, Cary, NC), and p < 0.05 was considered significant.

## Results

A total of 670,797 DM patients were followed up for a median 7.0 (mean 6.7 ± SD 1.3) years, and 22,019 (3.3%) were newly diagnosed with AF (incidence rate 4.9 per 1000 person-years). Bodyweight was measured in each subject, either 3 (74.0%), 4 (12.2%), or 5 times (13.8%). The baseline characteristics of the study population are shown in Table 1. The mean age was 57.8 years, and 65% were men. The mean index bodyweight was 66.5 kg, and the mean index BMI was 25.0 kg/cm^2^. Around two-thirds of the subjects were on oral anti-diabetic medication, and 8% were on insulin.

**Table 1:** Baseline characteristics of the study population according to bodyweight variability

	Total (n = 670,797)	Reference bodyweight variability group (Q1–4) (n = 536,459)	High bodyweight variability group (Q5) (n = 134,338)
Age, years	57.80 ± 11.47	57.55 ± 11.15	58.79 ± 12.65
30–39 years	45433 (6.77)	33665 (6.28)	11768 (8.76)
40–64 years	436588 (65.08)	359809 (67.07)	76779 (57.15)
≥ 65 years	188776 (28.14)	142985 (26.65)	45791 (34.09)
Male	435633 (64.94)	357633 (66.67)	78000 (58.06)
Number of oral anti-diabetic medication^a^			
0	226812 (33.81)	186815 (34.82)	39997 (29.77)
1	139300 (20.77)	110250 (20.55)	29050 (21.62)
2	195051 (29.08)	154610 (28.82)	40441 (30.1)
3 or more	109634 (16.34)	84784 (15.80)	24850 (18.50)
Insulin use	51712 (7.71)	36711 (6.84)	15001 (11.17)
Hypertension	381019 (56.80)	303865 (56.64)	77154 (57.43)
Dyslipidemia	255584 (38.10)	204328 (38.09)	51256 (38.15)
Decreased renal function (eGFR < 60 mL/min)	76556 (11.43)	58987 (11.01)	17569 (13.09)
Smoking status			
Non-smoker	364796 (54.38)	286199 (53.35)	78597 (58.51)
Ex-smoker	139872 (20.85)	115510 (21.53)	24362 (18.13)
Current smoker	166129 (24.77)	134750 (25.12)	31379 (23.36)
Drinking status			
Non-drinker	371857 (55.44)	287962 (53.68)	83895 (62.45)
Mild drinker	236249 (35.22)	196699 (36.67)	39550 (29.44)
Heavy drinker	62691 (9.35)	51798 (9.66)	10893 (8.11)
Regular exercise	159100 (23.72)	130269 (24.28)	28831 (21.46)
Low income	157722 (23.51)	124951 (23.29)	32771 (24.39)
Health examination			
Body weight, kg	66.52 ± 11.29	67.03 ± 10.92	64.49 ± 12.45
Height, cm	162.95 ± 9.07	163.27 ± 8.92	161.66 ± 9.53
Body mass index, kg/m^2^	24.97 ± 3.14	25.07 ± 3.01	24.57 ± 3.59
Waist circumference, cm	85.44 ± 8.14	85.65 ± 7.94	84.63 ± 8.88
SBP, mmHg	129 ± 15	129 ± 15	128 ± 16
DBP, mmHg	79 ± 9	79 ± 10	78 ± 10
Fasting glucose, mg/dL	143 ± 43	142 ± 41	144 ± 50
Total cholesterol, mg/dL	195.54 ± 40.63	195.92 ± 40.28	194.04 ± 41.95
HDL-cholesterol, mg/dL	51.63 ± 21.34	51.47 ± 21.35	52.28 ± 21.26
LDL-cholesterol, mg/dL	110.73 ± 44.88	110.85 ± 44.70	110.23 ± 45.58
Triglyceride^b^, mg/dL	146.24 (146.04–146.44)	148.26 (148.03–148.49)	138.45 (138.02–138.87)
Weight variability indices			
SD, kg	2.02 ± 1.49	1.49 ± 0.71	4.15 ± 1.85
CV, %	3.07 ± 2.26	2.23 ± 1.02	6.41 ± 2.72
VIM, %	1.98 ± 1.45	1.44 ± 0.65	4.11 ± 1.74
ASV, kg	2.31 ± 1.81	1.73 ± 0.95	4.63 ± 2.47

Q, quintile; S(D)BP, Systolic (Diastolic) blood pressure; HDL, high-density lipoprotein; LDL, low-density lipoprotein; eGFR, estimated glomerular filtration rate by Modification of Diet in Renal Disease equation; SD, standard deviation; CV, coefficient of variation; VIM, variability independent of mean; ASV, average successive variability

^a^Of 6 types of oral medication: sulfonylurea, metformin, meglitinides, thiazolidinediones, DPP4 inhibitor, alpha-glucosidase inhibitor

^b^Geometric mean (95% CI) due to data not following normal distribution

### High bodyweight variability and risk of atrial fibrillation

When the study population was categorized by deciles (D) of bodyweight variability, with D1 (lowest variability) as reference, there was no significant difference in AF risk among D1-8, while D9 showed 11% and D10 showed 20% increased risk for incident AF, respectively, in multivariable-adjusted analysis (Additional file 1: Fig. S1). Therefore, we defined “high bodyweight variability” as the top 20% or the highest quintile (Q) of variability (Q5) with a VIM cutoff of 2.75, and the rest (Q1–4) as “reference bodyweight variability.”

Subjects with high bodyweight variability were slightly older (58.8 vs. 57.6 years), less likely to be male, had lower bodyweight (64.5 vs. 67.0 kg) and lower BMI (24.6 vs. 25.0 kg/m^2^), a higher proportion of hypertension and decreased renal function, and more advanced DM with higher fasting glucose and a greater number of oral anti-diabetic medication or insulin use (Table 1). They were also less likely to smoke or drink or exercise regularly, and more likely to have a lower income. The mean absolute bodyweight difference between visits (ASV) was 4.63 kg for the high bodyweight variability group and 1.73 kg for the reference variability group.

**Fig. 2:**
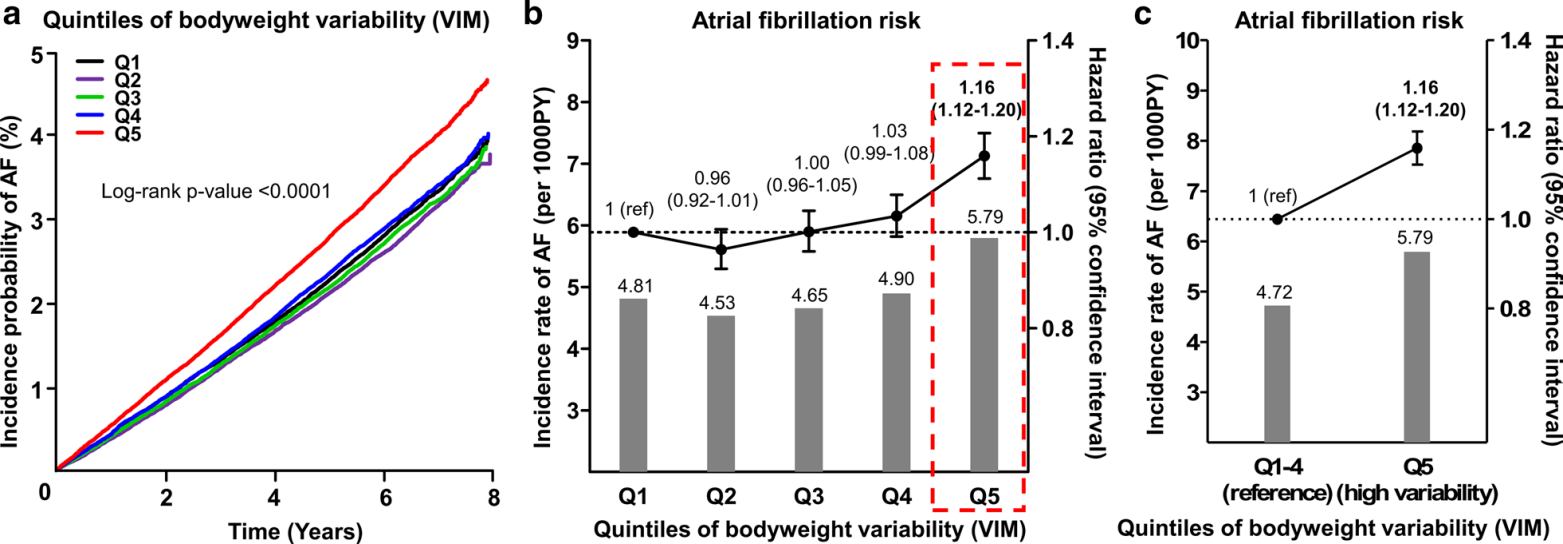
Atrial fibrillation risk was increased in the highest quintile of bodyweight variability. **a, b** Subjects in the highest quintile of bodyweight variability (Q5) showed significantly higher incidence and risk of AF compared to Q1–4, while there was no significant difference in AF risk among Q1–4. **c** AF risk was 16% increased in high bodyweight variability compared to reference bodyweight variability. Bar graphs represent incidence rates per 1000 person-years with scales on the left. Line graphs with error bars represent the hazard ratios with 95% confidence intervals for atrial fibrillation development with scales on the right. Hazard ratios were adjusted for baseline body mass index, age, sex, smoking, drinking, exercise, low income, hypertension, dyslipidemia, number of oral anti-diabetic medication, insulin use, duration of diabetes, and fasting glucose. VIM, variability independent of mean; AF, atrial fibrillation; Q, quintile

Subjects in the highest quintile of bodyweight variability (Q5) showed a significantly higher incidence of AF compared to Q1–4, while there was no significant difference in AF incidence among Q1–4 (Fig. 2a, b). In the multivariable-adjusted analysis, subjects with high bodyweight variability had a higher risk of AF development (HR 1.16, 95% CI 1.12–1.20) compared to those with reference bodyweight variability (Fig. 2c).

### Subgroup analyses

**Fig. 3:**
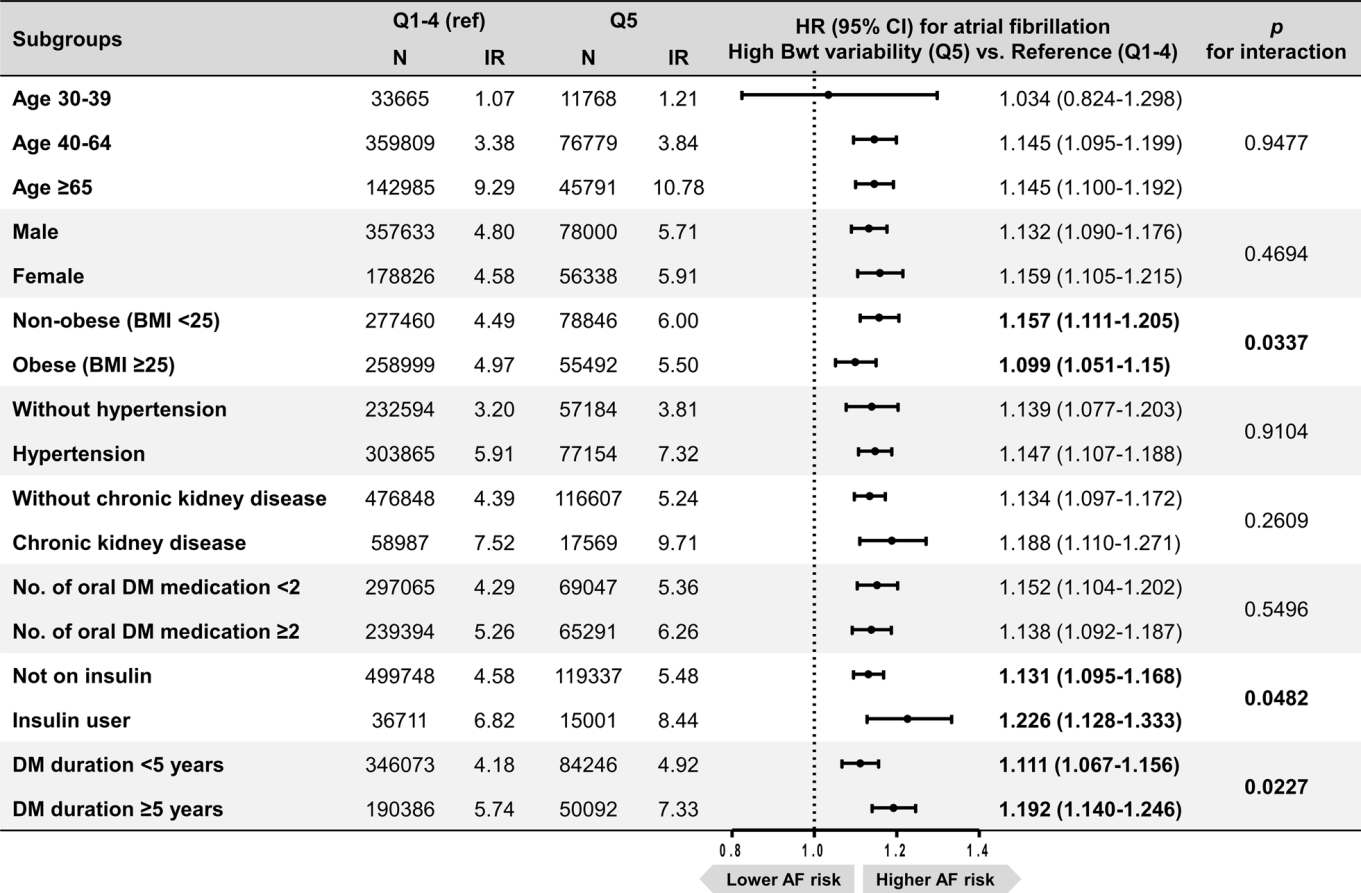
Subgroup analyses for atrial fibrillation risk according to bodyweight variability. Incidence rates (IR) per 1000 person-years. Hazard ratios (HR) were adjusted for baseline body mass index, age, sex, smoking, drinking, exercise, low income, hypertension, dyslipidemia, number of oral anti-diabetic medication, insulin use, duration of diabetes, and fasting glucose. Q, quintile; Bwt, bodyweight; BMI, body mass index; DM, diabetes mellitus

Incidence rates of AF were higher for subjects with high bodyweight variability in all subgroups by age strata, sex, presence of obesity, hypertension, or chronic kidney disease, number of oral anti-diabetic medication, insulin use, and DM duration. In all subgroup analyses, high bodyweight variability was consistently associated with AF development, except for similar AF risk in the young-age subgroup (Fig. 3). The association between high bodyweight variability and AF was significantly stronger in non-obese patients (BMI < 25 kg/m^2^) and those with more advanced diabetes, i.e. those on insulin or with a DM duration of greater than 5 years (all *P* for interaction < 0.05).

### Bodyweight variability and AF risk according to baseline BMI

Incidence rates and HRs for AF development showed a U-shaped relationship with baseline BMI, with the highest risk for AF in the underweight group (HR 1.25, 95% CI 1.10–1.42), and the lowest risk for AF in the overweight group (HR 0.93, 95% CI 0.89–0.98), in multivariable analysis with the normal weight group as reference (Additional file 1: Fig. S2A). AF risk was not significantly increased in obese DM patients, though there was a tendency for higher AF risk in obese stage II patients (BMI ≥ 30 kg/m^2^).

**Fig. 4:**
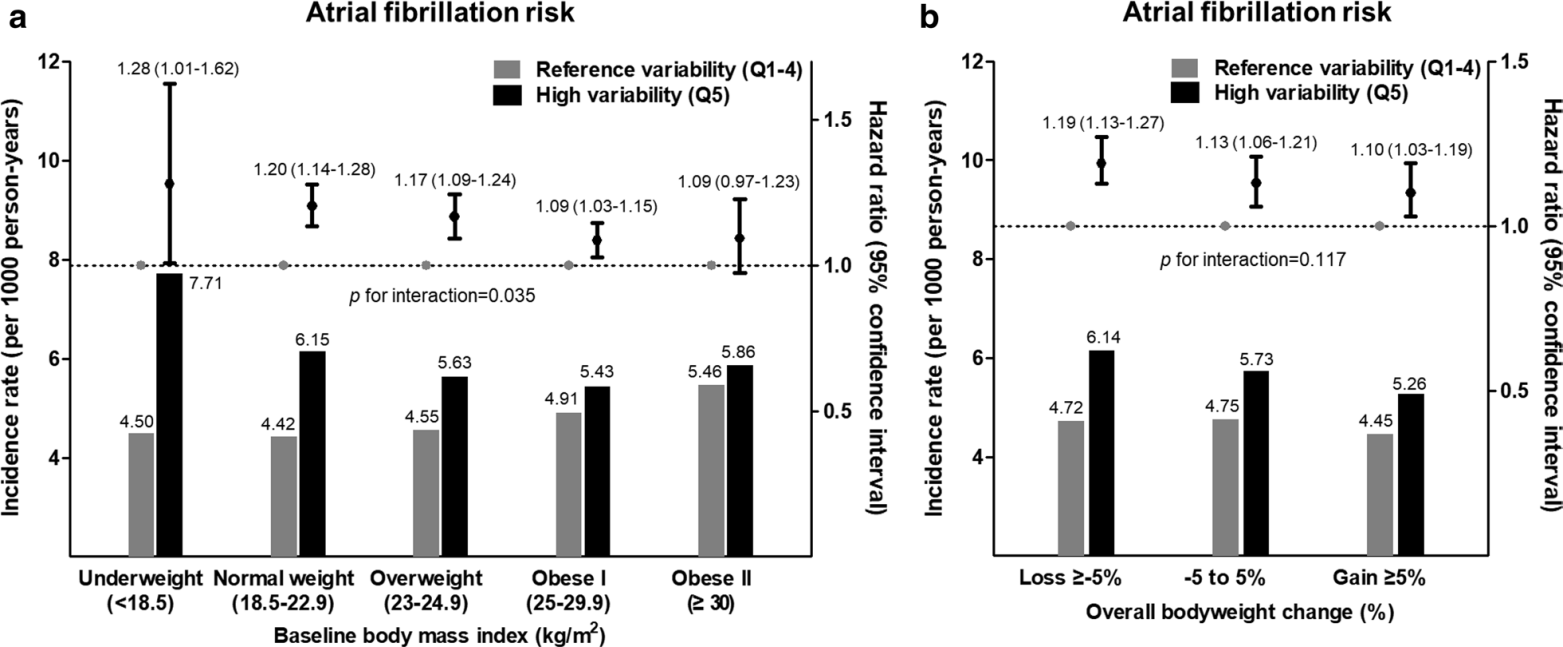
Atrial fibrillation risk according to bodyweight variability, stratified by **a** baseline body mass index and **b** overall bodyweight change. Bar graphs represent incidence rates per 1000 person-years with scales on the left. Line graphs with error bars represent the hazard ratios with 95% confidence intervals for atrial fibrillation development with scales on the right. Hazard ratios were adjusted for baseline body mass index, age, sex, smoking, drinking, exercise, low income, hypertension, dyslipidemia, number of oral anti-diabetic medication, insulin use, duration of diabetes, and fasting glucose. Q, quintile

AF incidence in subjects with reference bodyweight variability showed a proportional increase with BMI, with the highest AF incidence in the obese group. In contrast, AF incidence in subjects with high bodyweight variability generally increased with lower BMI, with the highest incidence rate of AF in the underweight group (Fig. 4a). After multivariable adjustment, high bodyweight variability was significantly associated with AF development in underweight, normal weight, overweight, and obese stage I groups. There was a trend of higher AF risk with higher bodyweight variability in the obese stage II group. The association of high bodyweight variability with AF became greater in magnitude with lower BMI (*P* for interaction = 0.035), and underweight subjects showed a higher risk of AF with high bodyweight variability (HR 1.28, 95% CI 1.01–1.62). Underweight DM patients with high bodyweight variability showed the highest incidence rate (7.71 per 1000 person-years) and the highest risk for incident AF (HR 1.51, 95% CI 1.29–1.78), compared to DM patients with normal weight and reference bodyweight variability (Additional file 1: Table S3).

### Bodyweight variability and AF risk according to the overall weight change

Incidence rate and HR for AF development were highest in subjects with weight loss (HR 1.11, 95% CI 1.08–1.15), and similar between subjects with stable weight or weight gain, in multivariable analysis with subjects with stable weight as reference (Additional file 1: Fig. S2B). When stratified by baseline BMI groups, this trend of higher AF risk with only weight loss was consistent in overweight and obese patients. In normal-weight or underweight patients, there was a tendency for higher AF risk with both weight gain and weight loss (Additional file 1: Table S4).

Subjects with high bodyweight variability showed a higher incidence and risk for AF compared to those with reference variability in all weight change groups (Fig. 4b). This trend was consistent after stratification by BMI into non-obese (BMI < 25) and obese (BMI ≥ 25) subjects (Additional file 1: Fig. S3); the strength of the association was greater in the non-obese subjects, which was observed in the subgroup analysis. DM patients with weight loss and high bodyweight variability showed the highest incidence rate (6.14 per 1000 person-years) and the highest risk for incident AF (HR 1.20, 95% CI 1.17–1.27), compared to patients with stable weight and normal bodyweight variability (Additional file 1: Table S3).

### Sensitivity analyses

Sensitivity analyses with other indices of variability, i.e. SD, CV, and ASV, also demonstrated a consistent association between high bodyweight variability and risk of AF development (Additional file 1: Table S5). When divided by quintiles, there was a steep increase in AF risk for Q5 compared to the lower 4 quintiles (Q1–4). In line with VIM results, high bodyweight variability (Q5) by SD, CV, and ASV was associated with an 18%, 15%, and 17% increase in hazard for incident AF, respectively, compared to reference bodyweight variability (Q1–4).

Glycemic variability was defined similarly to bodyweight variability from measurements of fasting glucose levels at health examinations done 3–5 times over a 5-year period. To note, while this method represents longer-term fasting glucose variability, it does not reflect short-term oscillations in glucose throughout the day or postprandial hyperglycemia. Exploratory analysis further adjusting for fasting glycemic variability showed similar results to the main analysis (Additional file 1: Table S6, S7). When the study population was divided into deciles by glycemic variability, there was no meaningful increase in AF risk with high glycemic variability (Additional file 1: Table S8).

## Discussion

The principal findings of the current study can be summarized as followings: (1) in a large nationwide population-based cohort of DM patients, risk of incident AF increased in those with the top 20% of bodyweight variability; these patients with high bodyweight variability showed a 16% higher risk of AF development compared to the rest of the population independent of traditional cardiovascular risk factors and baseline BMI; (2) high bodyweight variability was associated with AF development regardless of baseline BMI or direction of overall weight change; (3) this association was greater in magnitude in patients with lower BMI; underweight patients with high bodyweight variability showed the highest risk for AF development; and (4) the association between high bodyweight variability and AF development was consistent in subgroup analyses and was stronger in those with more advanced DM, i.e. those on insulin or with a DM duration of greater than 5 years.

To the best of our knowledge, this is the first study reporting the association of bodyweight variability and the risk of AF in patients with type 2 DM.

### Bodyweight variability as a risk factor for AF in DM

Weight change has been associated with AF development and burden. Obesity predisposes to a larger number of prolonged AF episodes in the early postoperative period after cardiac surgery [24]. In obese patients, bariatric surgery with forced weight loss was associated with significantly less incidence of AF compared to medically managed counterparts [6, 25]. In patients with established AF, weight reduction has been associatied with lower burden of AF and symptom severity [7, 8, 26].

Weight control is highly recommended to DM patients, but dieting and intentional weight control often result in weight fluctuation. Weight cycling, or bodyweight variability, has been associated with metabolic alterations, cardiovascular events, and death in the general population and patients with coronary disease [11, 12, 27]. We previously demonstrated that bodyweight variability was an independent predictor of AF in the general population [15]. Increasing bodyweight variability is associated with higher risks of coronary events, heart failure, and death in type 2 DM patients [13, 14]. Herein, we demonstrated that high bodyweight variability was independently associated with AF development in patients with type 2 DM.

Bangalore et al. showed that greater bodyweight variability was continuously associated with the risk of coronary or cardiovascular events and death in DM patients, and that this association was continuous over the whole range of bodyweight variability and was more pronounced in overweight and obese patients [13]. In this study, we found that the risk of AF development was increased in the top 20% of bodyweight variability, and the association between bodyweight variability and AF development was more pronounced in underweight DM patients after adjustment for baseline BMI.

Of note, there are some differences in the association between bodyweight and AF in the diabetic and the general population [16]. While the incidence of AF was highest for the most obese subjects in the general population (3.39 per 1000 person-years), it was highest in the diabetic population amongst the underweight subjects (5.89 per 1000 person-years). The association between bodyweight variability and AF development was greater in magnitude with lower BMI in both populations, but was more pronounced in the diabetic population (HR for high bodyweight variability vs. reference bodyweight variability in the underweight group: general population, 1.16, 95% CI 1.08–1.24; diabetic population, 1.28, 95% CI 1.01–1.62).

In patients with type 2 DM, overweight and obesity, and weight gain are synergistically associated with increased risk of AF [4, 5]. On the other hand, underweight and weight loss were associated with increased risk for AF [4, 28–30], even after adjustment for baseline comorbidities. In our study, while there was a trend for increased risk of AF with obesity, AF risk was also increased with underweight and weight loss, after adjustment for baseline BMI, comorbidities, and lifestyle variables. One explanation is that while intentional weight loss is beneficial, unintentional weight loss may be related to hidden chronic diseases and cachexia, which can increase AF susceptibility. Other potential hypotheses for the link between underweight/weight loss and AF include loss of muscle mass, malnutrition, and high adiponectin levels [30–33]. Furthermore, weight loss may be a part of weight cycling, and weight regain has been associated with rapid adipose tissue accumulation with a higher concentration of adipocytes due to altered metabolism favoring lipid storage [27], which act as a source of pro-inflammatory cytokines disposing to cardiac remodeling and AF [34].

### Patholophysiological implications of bodyweight variability

Diabetes is associated with cardiac fibrosis, electrical remodeling, and a higher risk of AF, related to oxidative stress, inflammation, and glycemic fluctuations [35]. Insulin resistance is associated with atrial structural remodeling and abnormal intracellular calcium homeostasis, contributing to increased AF susceptibility [36]. Obesity is associated with dilatation of the cardiac chambers, neurohormonal activation, and electroanatomical remodeling of the atria with fibrofatty infiltration from the epicardial fat [37, 38]. Both diabetes and obesity cause expansion of the epicardial adipose tissue, the source of proinflammatory adipocytokines which can cause microvascular dysfunction and cardiac fibrosis, providing the substrate for AF [34]. Weight fluctuation resulted in extensive fibro-fatty infiltrations and myolysis of the atria that persisted after weight normalization in sheep [39]. Weight regain is associated with with rapid adipose tissue growth and hyperplasia [27], and weight cycling has also been associated with glucose intolerance and the development of diabetes [40, 41]. Therefore, the combination of diabetes and obesity, especially weight cycling, can be synergistic in the development of AF. Glucose fluctuations have also been associated with oxidative stress and cardiac fibrosis [42, 43], possibly increasing risk of AF development [44]. In our study, bodyweight variability was associated with incident AF independent of fasting glucose variability.

Interestingly, high bodyweight variability was more strongly associated with AF development with advanced diabetic stage, suggesting that the pro-arrhythmic effects of bodyweight fluctuation and DM may be synergistic. Weight cycling may also interfere with the beneficial effects of weight loss and could be related to the futility of intervention focusing on weight loss in reducing cardiovascular events [45]. Indeed, our study suggests that weight fluctuation can be harmful and should be avoided when possible in weight control regimens for DM patients.

### Strengths and limitations

This study has the following strengths: (1) the large sample size of 670 thousand patients with type 2 DM; (2) the population-based design, minimizing selection bias; and (3) the long follow-up duration of median 7 years and the completeness of data, including all health care usage of subjects.

Several limitations should also be considered. First, the diagnosis of AF was based on claims data, and asymptomatic AF events without hospital visits could not be undetected. Also, the types of AF could not be specified as the database did not include electrocardiograms or information on the duration of arrhythmic episodes. Second, our analyses did not consider the competing risk of death, and the hazard ratios should be interpreted as ‘AF cause-specific’. Third, whether weight changes were intentional or unintentional are not known, and while unintentional weight change may be associated with underlying disease and adverse outcomes, intentional weight change may be beneficial. Fourth, we did not have available information on possible confounding factors such as baseline cancer. Fifth, while we suggest a synergistic relationship between DM and bodyweight variability in the process of AF development, the background mechanisms warrant further research. Also, the benefit of optimal weight control regimens minimizing weight fluctuation in DM patients need to be explored in further trials.

## Conclusions

In a large cohort of patients with type 2 DM, high variability in bodyweight was associated with AF development, independently of traditional cardiovascular risk factors and baseline BMI. This association was stronger in underweight patients and with advanced diabetic stage.

## Supplementary information

**Additional file 1.** Online only supplement.

## Data Availability

The datasets generated and analyzed during the current study are available on request at the Korea National Health Insurance Sharing Service hompage (nhiss.nhis.co.kr), to researchers approved by their institutional review boards.

## Abbreviations

AF: Atrial fibrillation
DM: Diabetes mellitus
ICD-10: International Statistical Classification of Diseases, Tenth Revision
VIM: Variability independent of the mean
SD: Standard deviation
CV: Coefficient of variation
ASV: Average successive variability
BMI: Body mass index
Q: Quintile
D: Decile
