# Supplementary material for: High variability in bodyweight is associated with an increased risk of atrial fibrillation in patients with type 2 diabetes mellitus: a nationwide cohort study
Source: Cardiovasc Diabetol. 2020 Jun 13;19:78. doi: 10.1186/s12933-020-01059-8 (PMC7293783; doi:10.1186/s12933-020-01059-8)
Supplement: Supplementary file 1 — Additional file 1. Online only supplement. [file 12933_2020_1059_MOESM1_ESM.docx]

**Online-Only Supplement**

1. **Additional file 1: Tables 1-8**
2. **Additional file 1: Figures 1-3**

**Additional file 1: Table 1. Definitions of comorbidities and outcomes.**

|  | ICD-10 codes with additional definitions | | Health examinations results |
| --- | --- | --- | --- |
| Comorbidities |  |  |  |
| Hypertension | I10-I13, I15 | Admission ≥1 or outpatient clinic ≥2, and prescription of anti-hypertensive drug (thiazide, loop diuretics, aldosterone antagonist, alpha-/beta-blocker, calcium-channel blocker, angiotensin-converting enzyme inhibitor, angiotensin II receptor blocker) | Systolic blood pressure ≥140 mmHg or diastolic blood pressure ≥90 mmHg |
| Dyslipidemia | E78 | Admission or outpatient department≥1, and prescription of lipid-lowering medication (statin, ezetimibe, fenofibrate) | Fasting total cholesterol ≥240 mg/dL |
| Outcomes |  |  |  |
| Atrial fibrillation | I48 | Admission or outpatient clinic ≥1 Exclusion of rheumatic mitral stenosis (I05.0, I05.2, I05.9), prosthetic heart valves (Z95.2-Z95.4) |  |

eGFR, estimated glomerular filtration rate by Modification of Diet in Renal Disease equation

**Additional file 1: Table 2. Definitions of lifestyle behaviors.**

|  | Category | Definition |
| --- | --- | --- |
| Smoking status | Non-smoker | Never-smoker or total lifetime smoking history <5 packs (100 cigarettes) |
|  | Ex-smoker | Total lifetime smoking history ≥5 packs (100 cigarettes) and quit smoking |
|  | Current smoker | Total lifetime smoking history ≥5 packs (100 cigarettes) and currently smoking |
| Drinking status | Non-drinker | Daily alcohol intake = 0 |
|  | Mild drinker | Alcohol intake <30g/day (and more than 0) |
|  | Heavy drinker | Alcohol intake ≥30g/day |
| Regular exercise |  | At least 150 minutes per week of moderate-intensity exercise or at least 60 minutes per week of vigorous-intensity exercise   - Moderate-intensity exercise: Exercise requiring a little more exertion or causing a little more breathlessness than usual - Vigorous-intensity exercise: Exercise requiring much more exertion or causing much more breathlessness than usual |
| Low income |  | Lowest income quartile according to the income-based premiums in the qualification database + Recipients of special medical care coverage for the impoverished |

**Additional file 1: Table 3. Atrial fibrillation risk according to body weight variability, stratified by baseline body mass index and overall weight change, with normal weight or stable weight as reference.**

|  | Bodyweight variability | N | IR | Adjusted^*^ HR (95% CI) |
| --- | --- | --- | --- | --- |
| Baseline BMI |  |  |  |  |
| Underweight (BMI <18.5 kg/m^2^) | Q1-4 (ref.) | 4485 | 4.50 | 1.155 (0.963-1.385) |
|  | Q5 (high) | 3772 | 7.71 | 1.513 (1.287-1.779) |
| Normal weight (BMI 18.5-22.9 kg/m^2^) | Q1-4 (ref.) | 126622 | 4.42 | 1 (ref.) |
|  | Q5 (high) | 42903 | 6.15 | 1.214 (1.146-1.287) |
| Overweight (BMI 23-24.9 kg/m^2^) | Q1-4 (ref.) | 146353 | 4.55 | 0.953 (0.905-1.003) |
|  | Q5 (high) | 32171 | 5.63 | 1.113 (1.037-1.194) |
| Obese stage I (BMI 25-29.9 kg/m^2^) | Q1-4 (ref.) | 227392 | 4.91 | 0.992 (0.923-1.065) |
|  | Q5 (high) | 45549 | 5.43 | 1.074 (0.988-1.168) |
| Obese stage II (BMI ≥30 kg/m^2^) | Q1-4 (ref.) | 31607 | 5.46 | 1.115 (0.976-1.273) |
|  | Q5 (high) | 9943 | 5.86 | 1.219 (1.039-1.430) |
| Overall weight change |  |  |  |  |
| Weight loss (Loss ≥-5%) | Q1-4 (ref.) | 62965 | 4.72 | 1.013 (0.966-1.063) |
|  | Q5 (high) | 68936 | 6.14 | 1.202 (1.169-1.273) |
| Stable weight (<-5% to <5%) | Q1-4 (ref.) | 426120 | 4.75 | 1 (ref.) |
|  | Q5 (high) | 24380 | 5.73 | 1.129 (1.055-1.207) |
| Weight gain (Gain ≥ 5%) | Q1-4 (ref.) | 47374 | 4.45 | 0.986 (0.933-1.041) |
|  | Q5 (high) | 41022 | 5.26 | 1.081 (1.023-1.142) |

Incidence rate (IR) per 1,000 person-years. Hazard ratios (HR) were adjusted for baseline body mass index, age, sex, smoking, drinking, exercise, low income, hypertension, dyslipidemia, number of oral anti-diabetic medication, insulin use, duration of diabetes, and fasting glucose.

BMI, body mass index.

**Additional file 1: Table 4. Atrial fibrillation risk according to overall body weight change stratified by baseline body mass index.**

| BMI intervals | Bodyweight change | N | IR | Adjusted^*^ HR (95% CI) |
| --- | --- | --- | --- | --- |
| Underweight  (BMI <18.5 kg/m^2^) | Loss ≥-5% | 4289 | 7.05 | 1.191 (0.928-1.529) |
|  | <-5% to <5% | 3599 | 4.51 | 1 (ref.) |
|  | Gain ≥5% | 369 | 6.94 | 1.469 (0.866-2.491) |
| Normal weight  (BMI 18.5-22.9 kg/m^2^) | Loss ≥-5% | 52433 | 5.42 | 1.083 (1.021-1.149) |
|  | <-5% to <5% | 101920 | 4.53 | 1 (ref.) |
|  | Gain ≥5% | 15172 | 4.98 | 1.129 (1.026-1.241) |
| Overweight  (BMI 23-24.9 kg/m^2^) | Loss ≥-5% | 35953 | 5.26 | 1.102 (1.033-1.176) |
|  | <-5% to <5% | 122862 | 4.61 | 1 (ref.) |
|  | Gain ≥5% | 19709 | 4.62 | 1.043 (0.957-1.137) |
| Obese stage I (BMI 25-29.9 kg/m^2^) | Loss ≥-5% | 35507 | 5.43 | 1.112 (1.047-1.182) |
|  | <-5% to <5% | 194934 | 4.97 | 1 (ref.) |
|  | Gain ≥5% | 42500 | 4.71 | 0.973 (0.918-1.032) |
| Obese stage II (BMI ≥30 kg/m^2^) | Loss ≥-5% | 3719 | 6.10 | 1.167 (0.982-1.386) |
|  | <-5% to <5% | 27185 | 5.55 | 1 (ref.) |
|  | Gain ≥5% | 10646 | 5.36 | 0.986 (0.875-1.110) |

Incidence rate (IR) per 1,000 person-years. Hazard ratios (HR) were adjusted for baseline body mass index, age, sex, smoking, drinking, exercise, low income, hypertension, dyslipidemia, number of oral anti-diabetic medication, insulin use, duration of diabetes, and fasting glucose.BMI, body mass index.

**Additional file 1: Table 5. Sensitivity analyses with various bodyweight variability indices for risk of atrial fibrillation according to quintiles of bodyweight variability.**

| Bodyweight variability index | N | IR | Adjusted HR (95% CI) |
| --- | --- | --- | --- |
| VIM |  |  |  |
| Q1 | 134160 | 4.81 | 1 (ref.) |
| Q2 | 134451 | 4.53 | 0.964 (0.924-1.006) |
| Q3 | 133868 | 4.65 | 1.001 (0.960-1.045) |
| Q4 | 133980 | 4.90 | 1.034 (0.991-1.078) |
| Q5 | 134338 | 5.79 | 1.159 (1.112-1.207) |
| Q1-4 | 536459 | 4.72 | 1 (ref.) |
| Q5 | 134338 | 5.79 | 1.159 (1.122-1.196) |
| SD |  |  |  |
| Q1 | 110229 | 4.65 | 1 (ref.) |
| Q2 | 144746 | 4.50 | 0.987 (0.944-1.032) |
| Q3 | 147732 | 4.93 | 1.030 (0.986-1.076) |
| Q4 | 134092 | 4.88 | 1.053 (1.007-1.101) |
| Q5 | 133998 | 5.71 | 1.205 (1.153-1.258) |
| Q1-4 | 536799 | 4.74 | 1 (ref.) |
| Q5 | 133998 | 5.71 | 1.183 (1.146-1.221) |
| CV |  |  |  |
| Q1 | 134238 | 4.82 | 1 (ref.) |
| Q2 | 134209 | 4.50 | 0.964 (0.923-1.006) |
| Q3 | 134126 | 4.68 | 1.003 (0.962-1.047) |
| Q4 | 134161 | 4.90 | 1.036 (0.994-1.081) |
| Q5 | 134063 | 5.78 | 1.149 (1.103-1.198) |
| Q1-4 | 536734 | 4.73 | 1 (ref.) |
| Q5 | 134063 | 5.78 | 1.148 (1.112-1.185) |
| ASV |  |  |  |
| Q1 | 171792 | 4.68 | 1 (ref.) |
| Q2 | 112882 | 4.52 | 0.995 (0.953-1.038) |
| Q3 | 118069 | 4.56 | 1.055 (1.011-1.100) |
| Q4 | 135216 | 5.05 | 1.087 (1.044-1.130) |
| Q5 | 132838 | 5.84 | 1.204 (1.159-1.252) |
| Q1-4 | 537959 | 4.71 | 1 (ref.) |
| Q5 | 132838 | 5.84 | 1.166 (1.130-1.204) |

Incidence rate (IR) per 1,000 person-years. Hazard ratios (HR) were adjusted for baseline body mass index, age, sex, smoking, drinking, exercise, low income, hypertension, dyslipidemia, number of oral anti-diabetic medication, insulin use, duration of diabetes, and fasting glucose.Q, quintile (Q5 representing highest quintile); VIM, variability independent of mean; SD, standard deviation; CV, coefficient of variation; ASV, average successive variability.

**Additional file 1: Table 6. Sensitivity analysis with further adjustment with glycemic variability, for atrial fibrillation risk according to quintiles of bodyweight variability.**

| Bodyweight variability (VIM) | N | IR | Adjusted HR^*^ (95% CI) | Further adjusted HR^†^ (95% CI) |
| --- | --- | --- | --- | --- |
| Q1 | 134160 | 4.81 | 1 (ref.) | 1 (ref.) |
| Q2 | 134451 | 4.53 | 0.964 (0.924-1.006) | 0.964 (0.923-1.006) |
| Q3 | 133868 | 4.65 | 1.001 (0.960-1.045) | 1.001 (0.959-1.044) |
| Q4 | 133980 | 4.90 | 1.034 (0.991-1.078) | 1.032 (0.989-1.077) |
| Q5 | 134338 | 5.79 | 1.159 (1.112-1.207) | 1.156 (1.109-1.204) |
| Q1-4 | 536459 | 4.72 | 1 (ref.) | 1 (ref.) |
| Q5 | 134338 | 5.79 | 1.159 (1.122-1.196) | 1.156 (1.120-1.194) |

Incidence rate (IR) per 1,000 person-years.

^*^ Hazard ratios (HR) were adjusted for baseline body mass index, age, sex, smoking, drinking, exercise, low income, hypertension, dyslipidemia, number of oral anti-diabetic medication, insulin use, duration of diabetes, and fasting glucose.

^†^ Hazard ratios (HR) were adjusted for baseline body mass index, age, sex, smoking, drinking, exercise, low income, hypertension, dyslipidemia, number of oral anti-diabetic medication, insulin use, duration of diabetes, fasting glucose, and glycemic variability.

VIM, variability independent of mean; Q, quintile (Q5 representing the highest quintile).

**Additional file 1: Table 7. Sensitivity analysis with further adjustment with glycemic variability, for atrial fibrillation risk according to bodyweight variability stratified by baseline body mass index and overall weight change.**

|  | Bodyweight variability | N | IR | Adjusted^*^ HR (95% CI) | Further adjusted^†^ HR (95% CI) |
| --- | --- | --- | --- | --- | --- |
| Baseline BMI |  |  |  |  |  |
| Underweight  (BMI <18.5 kg/m^2^) | Q1-4 (ref.) | 4485 | 4.50 | 1 (ref.) | 1 (ref.) |
|  | Q5 (high) | 3772 | 7.71 | 1.279 (1.007-1.623) | 1.279 (1.008-1.624) |
| Normal weight  (BMI 18.5-22.9 kg/m^2^) | Q1-4 (ref.) | 126622 | 4.42 | 1 (ref.) | 1 (ref.) |
|  | Q5 (high) | 42903 | 6.15 | 1.204 (1.135-1.277) | 1.200 (1.131-1.273) |
| Overweight  (BMI 23-24.9 kg/m^2^) | Q1-4 (ref.) | 146353 | 4.55 | 1 (ref.) | 1 (ref.) |
|  | Q5 (high) | 32171 | 5.63 | 1.166 (1.093-1.244) | 1.161 (1.088-1.239) |
| Obese stage I  (BMI 25-29.9 kg/m^2^) | Q1-4 (ref.) | 227392 | 4.91 | 1 (ref.) | 1 (ref.) |
|  | Q5 (high) | 45549 | 5.43 | 1.085 (1.028-1.145) | 1.087 (1.030-1.148) |
| Obese stage II  (BMI ≥30 kg/m^2^) | Q1-4 (ref.) | 31607 | 5.46 | 1 (ref.) | 1 (ref.) |
|  | Q5 (high) | 9943 | 5.86 | 1.092 (0.973-1.226) | 1.094 (0.974-1.228) |
| Overall weight change |  |  |  |  |  |
| Weight loss  (Loss ≥-5%) | Q1-4 (ref.) | 62965 | 4.72 | 1 (ref.) | 1 (ref.) |
|  | Q5 (high) | 68936 | 6.14 | 1.193 (1.125-1.266) | 1.191 (1.122-1.263) |
| Stable weight  (<-5% to <5%) | Q1-4 (ref.) | 426120 | 4.75 | 1 (ref.) | 1 (ref.) |
|  | Q5 (high) | 24380 | 5.73 | 1.130 (1.057-1.209) | 1.129 (1.055-1.207) |
| Weight gain  (Gain ≥ 5%) | Q1-4 (ref.) | 47374 | 4.45 | 1 (ref.) | 1 (ref.) |
|  | Q5 (high) | 41022 | 5.26 | 1.103 (1.025-1.188) | 1.099 (1.021-1.184) |

Incidence rate (IR) per 1,000 person-years.

^*^ Hazard ratios (HR) were adjusted for baseline body mass index, age, sex, smoking, drinking, exercise, low income, hypertension, dyslipidemia, number of oral anti-diabetic medication, insulin use, duration of diabetes, and fasting glucose.

^†^ Hazard ratios (HR) were adjusted for baseline body mass index, age, sex, smoking, drinking, exercise, low income, hypertension, dyslipidemia, number of oral anti-diabetic medication, insulin use, duration of diabetes, fasting glucose, and glycemic variability.

BMI, body mass index; Q, quintile (Q5 representing the highest quintile).

**Additional file 1: Table 8. Atrial fibrillation risk according to glycemic variability.**

| Glycemic variability (VIM) | N | IR | Adjusted HR (95% CI) |
| --- | --- | --- | --- |
| D1 | 67080 | 5.13 | 1 (ref.) |
| D2 | 67086 | 5.17 | 1.038 (0.979-1.100) |
| D3 | 67073 | 4.92 | 1.013 (0.955-1.074) |
| D4 | 67084 | 5.06 | 1.056 (0.996-1.119) |
| D5 | 67075 | 4.73 | 1.002 (0.945-1.063) |
| D6 | 67082 | 4.95 | 1.050 (0.990-1.113) |
| D7 | 67078 | 4.87 | 1.051 (0.991-1.114) |
| D8 | 67080 | 4.98 | 1.084 (1.023-1.150) |
| D9 | 67080 | 4.82 | 1.061 (1.000-1.126) |
| D10 | 67079 | 4.69 | 1.048 (0.988-1.113) |

Incidence rate (IR) per 1,000 person-years. Hazard ratios (HR) were adjusted for baseline body mass index, age, sex, smoking, drinking, exercise, low income, hypertension, dyslipidemia, number of oral anti-diabetic medication, insulin use, duration of diabetes, and fasting glucose.

VIM, variability independent of mean; D, decile (D10 representing the highest decile).

**
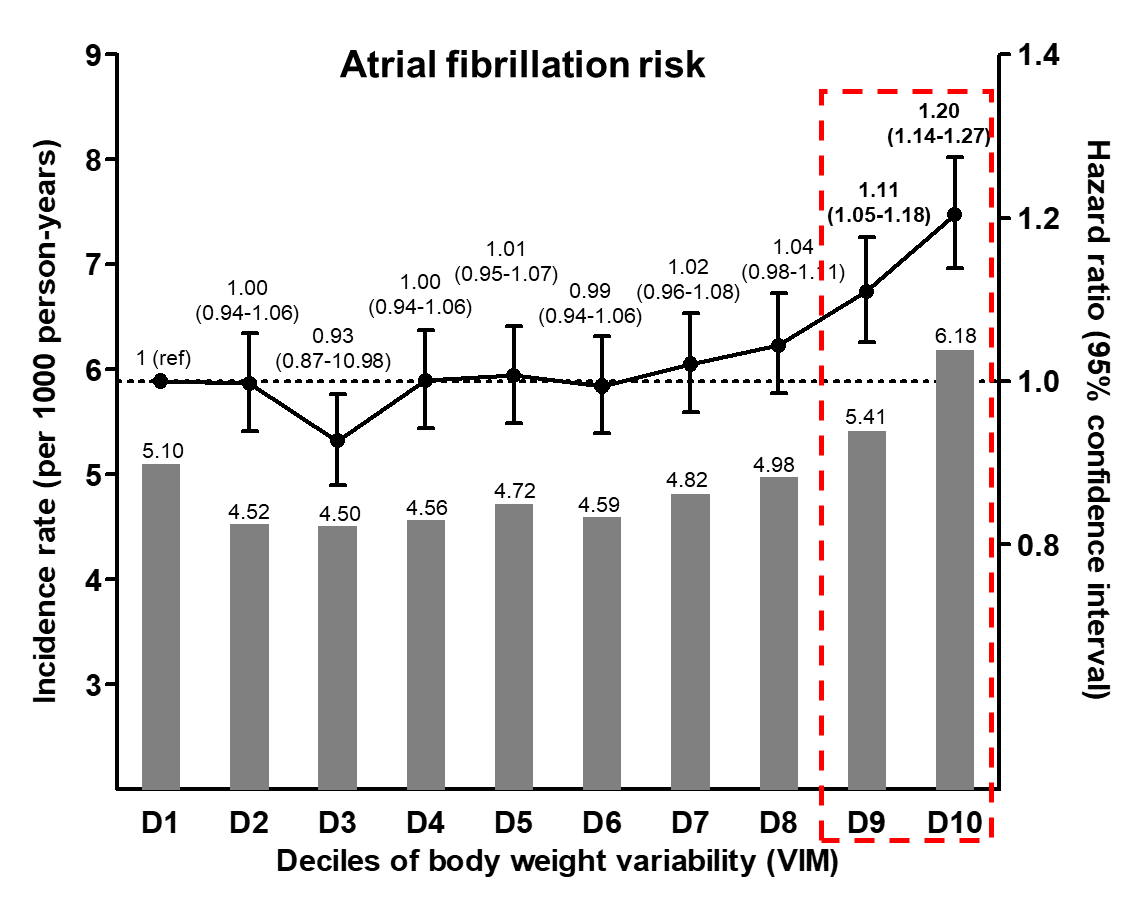
**

**Additional file 1: Figure 1. Atrial fibrillation risk according to deciles of bodyweight variability.**

Bar graphs represent incidence rates per 1,000 person-years with scales on the left. Line graphs with error bars represent the hazard ratios with 95% confidence intervals for atrial fibrillation development with scales on the right. Hazard ratios adjusted for baseline body mass index, age, sex, smoking, drinking, exercise, low income, hypertension, dyslipidemia, number of oral anti-diabetic medication, insulin use, and duration of diabetes.
VIM, variability independent of mean; D, decile.


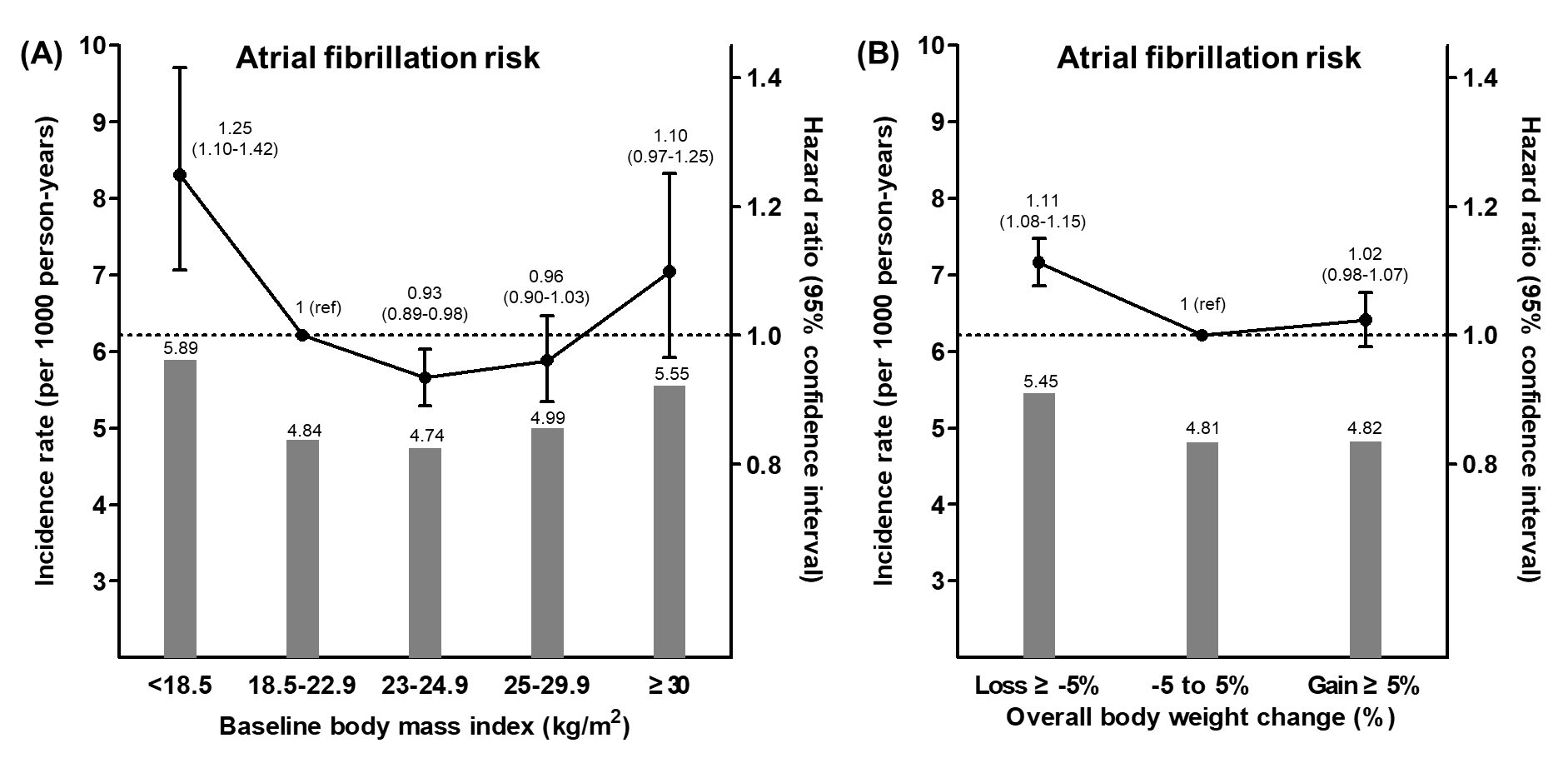


**Additional file 1: Figure 2. Atrial fibrillation risk according to (A) baseline body mass index or (B) overall weight change.**

Bar graphs represent incidence rates per 1,000 person-years with scales on the left. Line graphs with error bars represent the hazard ratios with 95% confidence intervals for atrial fibrillation development with scales on the right. Hazard ratios adjusted for baseline body mass index, age, sex, smoking, drinking, exercise, low income, hypertension, dyslipidemia, number of oral anti-diabetic medication, insulin use, and duration of diabetes.


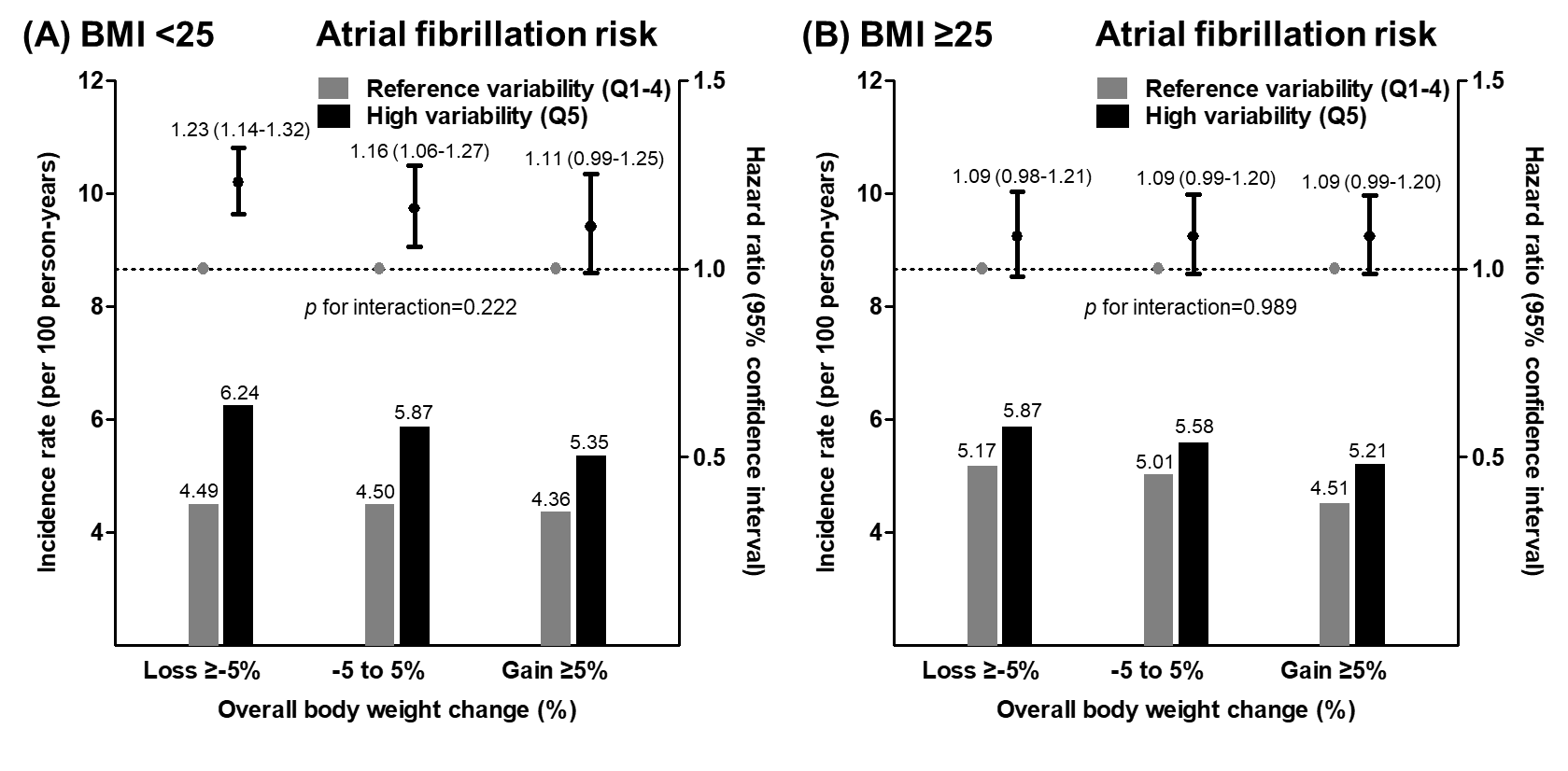


**Additional file 1: Figure 3. Atrial fibrillation risk according to body weight variability, stratified by overall body weight change in (A) non-obese and (B) obese subjects.**

Bar graphs represent incidence rates per 1,000 person-years with scales on the left. Line graphs with error bars represent the hazard ratios with 95% confidence intervals for atrial fibrillation development with scales on the right. Hazard ratios adjusted for baseline body mass index, age, sex, smoking, drinking, exercise, low income, hypertension, dyslipidemia, number of oral anti-diabetic medication, insulin use, and duration of diabetes.
BMI, body mass index; Q, quintile.
